# Pathological Outcomes of Patients With Advanced Renal Cell Carcinoma Who Receive Nephrectomy Following Immunotherapy

**DOI:** 10.1093/oncolo/oyad166

**Published:** 2023-06-27

**Authors:** Justine Panian, Ava Saidian, Kevin Hakimi, Archana Ajmera, William J Anderson, Pedro Barata, Stephanie Berg, Sabina Signoretti, Steven Lee Chang, Vincent D’Andrea, Daniel George, Hannah Dzimitrowicz, Talal El Zarif, Hamid Emamekhoo, Evan Gross, Deepak Kilari, Elaine Lam, Isabel Lashgari, Sarah Psutka, Grant P Rauterkus, Ahmed Shabaik, Bicky Thapa, Luke Wang, Nicole Weise, Kendrick Yim, Tian Zhang, Ithaar Derweesh, Rana R McKay

**Affiliations:** University of California San Diego, Department of Medicine, Division of Hematology-Oncology La Jolla, CA, USA; University of California San Diego, Department of Urology, La Jolla, CA, USA; University of California San Diego, Department of Urology, La Jolla, CA, USA; University of California San Diego, Department of Medicine, Division of Hematology-Oncology, La Jolla, CA, USA; Brigham and Women’s Hospital, Department of Pathology, Boston, MA, USA; Tulane University, Deming Department of Medicine, New Orleans, LA, USA; Loyola University Chicago, Department of Cancer Biology and Internal Medicine, Maywood, IL, USA; Brigham and Women’s Hospital, Department of Pathology, Boston, MA, USA; Brigham and Women’s Hospital, Division of Urology, Boston, MA, USA; Brigham and Women’s Hospital, Division of Urology, Boston, MA, USA; Duke Cancer Institute, Department of Medicine, Durham, NC, USA; Duke Cancer Institute, Department of Medicine, Durham, NC, USA; Dana-Farber Cancer Institute, Department of Medical Oncology, Boston, MA, USA; University of Wisconsin, Department of Medicine, Madison, WI, USA; The University of Washington, Department of Urology, Seattle, WA, USA; Medical College of Wisconsin, Department of Internal Medicine, Milwaukee, WI, USA; University of Colorado Cancer Center, Division of Medical Oncology, Aurora, CO, USA; San Diego State University, Department of Cell and Molecular Biology, San Diego, CA, USA; The University of Washington, Department of Urology, Seattle, WA, USA; Tulane University, Deming Department of Medicine, New Orleans, LA, USA; University of California San Diego, Department of Pathology, La Jolla, CA, USA; Medical College of Wisconsin, Department of Internal Medicine, Milwaukee, WI, USA; University of California San Diego, Department of Urology, La Jolla, CA, USA; University of California San Diego, Department of Medicine, Division of Hematology-Oncology La Jolla, CA, USA; Brigham and Women’s Hospital, Division of Urology, Boston, MA, USA; UT Southwestern, Department of Internal Medicine, Dallas, TX, USA; University of California San Diego, Department of Urology, La Jolla, CA, USA; University of California San Diego, Department of Medicine, Department of Urology, La Jolla, CA, USA

**Keywords:** renal cell carcinoma, immunotherapy, nephrectomy, metastatic, pathologic

## Abstract

**Background:**

Even though cytoreductive nephrectomy (CN) was once the standard of care for patients with advanced renal cell carcinoma (RCC), its role in treatment has not been well analyzed or defined in the era of immunotherapy (IO).

**Materials and Methods:**

This study analyzed pathological outcomes in patients with advanced or metastatic RCC who received IO prior to CN. This was a multi-institutional, retrospective study of patients with advanced or metastatic RCC. Patients were required to receive IO monotherapy or combination therapy prior to radical or partial CN. The primary endpoint assessed surgical pathologic outcomes, including American Joint Committee on Cancer (AJCC) staging and frequency of downstaging, at the time of surgery. Pathologic outcomes were correlated to clinical variables using a Wald-chi squared test from Cox regression in a multi-variable analysis. Secondary outcomes included objective response rate (ORR) defined by response evaluation criteria in solid tumors (RECIST) version 1.1 and progression-free survival (PFS), which were estimated using the Kaplan-Meier method with reported 95% CIs.

**Results:**

Fifty-two patients from 9 sites were included. Most patients were male (65%), 81% had clear cell histology, 11% had sarcomatoid differentiation. Overall, 44% of patients experienced pathologic downstaging, and 13% had a complete pathologic response. The ORR immediately prior to nephrectomy was stable disease in 29% of patients, partial response in 63%, progressive disease in 4%, and 4% unknown. Median follow-up for the entire cohort was 25.3 months and median PFS was 3.5 years (95% CI, 2.1-4.9).

**Conclusions:**

IO-based interventions prior to CN in patients with advanced or metastatic RCC demonstrates efficacy, with a small fraction of patients showing a complete response. Additional prospective studies are warranted to investigate the role of CN in the modern IO-era.

## Teaser

Despite advances in metastatic systemic therapy, the role of cytoreductive nephrectomy has not been formally evaluated in the immunotherapy era. Considering the limited data of patients receiving immunotherapy followed by cytoreductive nephrectomy, this study investigated the outcomes of patients of receiving immunotherapy who subsequently underwent cytoreductive nephrectomy.

**Implications for Practice**

This article reports the first, to the authors’ knowledge, real-world data on the pathologic outcomes of patients with advanced or metastatic RCC who received immunotherapy-based treatments followed by CN. The findings are highly clinically relevant, given the lack of data on pathologic outcomes with CN in the modern era. This analysis showed that immunotherapies are associated with pathological downstaging in 44% of patients and complete pathologic response in 13%. These findings will aid clinicians in understanding expectations for pathologic outcomes at CN post-immunotherapy.

## Introduction

Renal cell carcinoma (RCC) is a common disease in the US, with an estimated 79,000 new cases and 13,920 deaths in 2022.^1^ Of all patients with RCC, approximately 16% have advanced disease.^2^ The treatment paradigm for patients with locally advanced or metastatic RCC has dramatically changed over the past decade. Historically, upfront cytoreductive nephrectomy (CN) was considered the standard of care for patients with de novo metastatic disease. This was largely based on clinical trials that were conducted during the cytokine era, which demonstrated an improvement in overall survival with CN.^3^ During the targeted therapy era, the practice of upfront CN continued, supported by data derived primarily from large retrospective studies.^4,5^

In 2018, the international phase III CARMENA trial investigated metastatic clear cell patients with RCC who were candidates for CN to undergo nephrectomy and then receive sunitinib or receive sunitinib alone. This trial challenged the existing paradigm of upfront CN for patients with de novo metastatic RCC.^6^ The CARMENA trial demonstrated that sunitinib alone was non-inferior to sunitinib post-CN.^7^ While the CARMENA trial has been criticized for the high enrollment of patients with poor risk disease and higher tumor volume in addition to delays in accrual, the results have influenced the role of upfront CN in the modern era.^8,9^ These data were complimented by the international phase III SURTIME trial, which demonstrated that immediate CN limited exposure to systemic therapy and potentially compromised outcomes for patients with advanced disease, further supporting the role of upfront systemic therapy rather than initial CN in the metastatic patient population.^10^ In modern practice, CN continues to be used, albeit in carefully selected individuals based on performance status, disease burden, symptoms, and sites of metastasis.^11,12^ Currently, a large portion of patients with de novo metastatic disease initially receive systemic therapy prior to surgery.^13^

In parallel to changes in the surgical practice of advanced RCC, systemic treatment options for patients with treatment naïve advanced RCC have vastly improved. Multiple phase III clinical trials have demonstrated superior efficacy of contemporary therapies including improved response rates and overall survival with immunotherapy (IO) combinations compared to sunitinib alone for patients with metastatic clear cell RCC.^14-18^ Despite these revolutionary advancements in metastatic systemic therapy, the role of CN has not been formally evaluated in the immunotherapy era.^19^

Given limited data of patients receiving IO followed by CN, we designed a multi-institutional retrospective study to investigate the outcomes of patients of receiving IO therapy who subsequently underwent CN.

## Materials (Patients) and Methods

This was a multi-institutional, retrospective study that included 9 academic sites: Dana Farber Cancer Institute, Duke University, Loyola University Chicago, Medical College of Wisconsin, Tulane University, University of California San Diego, University of Colorado, University of Washington, and University of Wisconsin at Madison. The study was approved by the Institutional Review Boards at the respective institutions.

Eligible patients had locally advanced or metastatic RCC not suitable for upfront CN by local providers assessment. Due to the retrospective nature of this study, the specific rationale for why an individual was not suitable for upfront surgery was not available. Inclusion criteria required patients to have received at least one dose of an immune checkpoint inhibitor prior to receiving surgery (either a partial or radical CN). Eligible treatments included anti-program cell death protein 1(PD-1) or anti-programmed death-ligand 1 (PD-L1) agents administered as monotherapy or in combination with either cytotoxic T-lymphocyte-associated protein 4 (CTLA-4) or vascular endothelial growth factor (VEGF) targeted therapy. Patients were treated between May 3, 2016, and September 30, 2021.

Clinical data were collected in a consecutive fashion using an HIPAA-compliant data registry. Data on patient demographic, baseline characteristics, comorbidities, systemic therapy exposure, pre-and post-systemic therapy, pathological outcomes, and survival were extracted from the electronic medical records. The primary endpoint was pathological downstaging at time of post-IO, defined as a decline in T stage between the baseline clinical T stage and nephrectomy pathologic T stage, using the American Joint Committee on Cancer (AJCC) eighth edition. We used pathologic downstaging as this could be objectively and reproducible assessed in a retrospective series. Additionally, at the present time in kidney cancer, the field is lacking pathologic metrics such as minimum residual disease or residual cancer burden that are linked to pathologic outcomes. Secondary outcomes included progression-free survival (PFS) and objective response rate (ORR). PFS was the time from therapy initiation to radiographic progression, as determined by RECIST version 1.1 principles by local investigator assessment, or last follow-up; whichever came first. PFS was calculated for the therapy received immediately preceding CN. ORR was captured via RECIST 1.1 principles per investigator assessment. Even though all patients had their pathology confirmed by the surgical pathology report, nephrectomy specimens of patients who experienced a complete response of the primary tumor underwent repeat institutional pathologic confirmation by a genitourinary pathologist at the participating institutions.

Baseline characteristics were tabulated with categorical variables reported as numbers and percents and continuous variables reported as medians and ranges. The baseline timepoint was defined as the initiation of the line of systemic therapy that immediately preceded the CN. PFS was determined using the Kaplan-Meier method with reported 95% CIs and was included in the analysis for descriptive purposes alone to describe the cohort evaluatedGiven limited number of deaths, overall survival was not reported.

## Results

### Patient and Disease Characteristics

A total of 52 patients were included in this study across 9 institutions. Baseline characteristics are detailed in Table 1. The median age of patients was 63 years, the majority were male (65%), and 27% of patients were non-White. The predominant histology was clear cell (81%), 10% had some degree of sarcomatoid differentiation, and 10% demonstrated rhabdoid differentiation. Most patients presented with de novo distant metastases (85%) and 23% (*n* = 12) had bone and 23% (*n* = 12) had liver metastases at diagnosis. Tumor inferior vena cava (IVC) involvement prior to systemic therapy was present in 7 patients (13%): 6% (*n* = 3) of patients had involvement less than 2 cm above the renal vein, 4% (*n* = 2) of patients had involvement above the hepatic veins but below the diaphragm, and 4% (*n* = 2) of patients had involvement above the diaphragm. At the time of therapy initiation prior to nephrectomy, 65% of patients were treatment naïve (*n* = 34), 29% had received one prior line of therapy (*n* = 15), and 6% had received two prior lines of systemic therapy (*n* = 3). International Metastatic RCC Database Consortium (IMDC) risk, calculated at the start of systemic therapy immediately preceding nephrectomy, demonstrated that 6% of patients had favorable (*n* = 3) risk disease, 60% intermediate (*n* = 31) risk, 25% poor (*n* = 13) risk, and 10% unknown (*n* = 5) (Table 2).

**Table 1.** Baseline characteristics.

*Age (years)*	
Median	63
*Gender*	
Male	34 (65%)
Female	18 (35%)
*Race*	
White	38 (73%)
Black or African American	3 (6%)
Asian	4 (8%)
Native American/Pacific Islander	0 (0%)
Other or Mixed Race	7 (13%)
*Ethnicity*	
Hispanic	6 (12%)
Non-Hispanic	46 (88%)
*Pathology*	
Clear Cell RCC	34 (81%)
Papillary RCC	3 (6%)
Unclassified RCC	4 (8%)
Collecting Duct RCC	1 (2%)
XP Translocation	2 (4%)
De Novo Metastatic Disease	44 (85%)
Sarcomatoid Differentiation	5 (10%)
Rhabdoid Differentiation	5 (10%)
*Baseline metastases*	
Liver	12 (23%)
Bone	13 (25%)
Lymph node	20 (38%)
Lung	30 (58%)
*IMDC risk*	
Favorable	3 (6%)
Intermediate	31 (60%)
Poor	13 (25%)
Unknown	5 (10%)

**Table 2.** Imaging and pathologic response to IO.

Pre-nephrectomy RECIST 1.1		
	Systemic disease	Primary tumor
Progressive disease	2 (4%)	5 (10%)
Stable disease	15 (29%)	21 (40%)
Partial response	33 (63%)	22 (42%)
Unknown	2 (4%)	4 (8%)

Pathologic outcomes		
	Baseline T stage	Pathologic T stage
T0	0 (0%)	7 (13%)
T1	5 (10%)	8 (15%)
T2	14 (27%)	6 (12%)
T3	19 (37%)	27 (52%)
T4	13 (25%)	4 (8%)
Unknown	1 (2%)	0 (0%)

### Treatment Exposure

All patients received at least one line of IO prior to nephrectomy. The line of therapy that immediately preceded surgery included PD-1/CTLA-4 inhibitors (44%, *n* = 23), PD-1/PD-L1 monotherapy (25%, *n* = 13), PD-1/PD-L1 in combination with VEGF tyrosine kinase inhibitor (TKI) (23%, *n* = 12), and VEGF TKI monotherapy (8%, *n* = 4, Table 3). This is tabulated in Table 3. For the patients that are specified as receiving VEGF TKI monotherapy, they received a line of IO prior to the line of therapy immediately preceding nephrectomy. But for the purpose of study analysis, it was easiest to focus on this line of therapy immediately preceding surgery. The median duration of systemic therapy treatment prior to CN was 8.1 months (95% CI, 5.97-11.18). Following CN, 60% of patients continued the same systemic therapy after surgery (*n* = 31), 17% of patients switch to a different therapy after surgery (*n* = 9), and 23% of patients discontinued all systemic therapy (*n* = 12). Of the patients that discontinued all systemic therapy post-CN, 42% had evidence of distant metastases at therapy initiation (*n* = 5). Of the patients who continued the same treatment following CN, the median duration of therapy post-CN was 7.4 months (95% CI 4.4-10.8).

**Table 3.** Treatment data.

Type of therapy received prior to nephrectomy	
IO Monotherapy	13 (25%)
PD-L1 + CTLA-4	23 (44%)
IO + VEGF	12 (23%)
VEGF targeted alone	4 (8%)
Median treatment time (months)	
Line immediately preceding nephrectomy	8.1
Post-nephrectomy	7.4

### Pathologic Response

**Figure 1.**
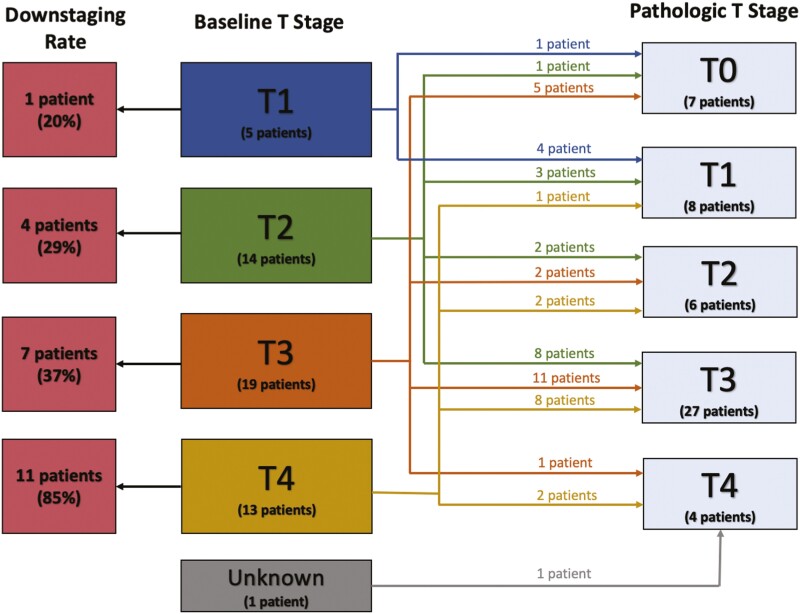
Degree of pathological downstaging (*n* = 52).

Overall, 44% of tumors demonstrated pathologic downstaging when comparing baseline clinical T stage (cT) and pathological T stage (ypT) at CN. We categorized the degree of pathological downstaging by baseline T stage, which is shown in Table 4 and illustrated in Fig. 1. Downstaging occurred in 20% of patients with cT1 (*n* = 5), 29% of cT2 (*n* = 14), 37% of cT3 (*n* = 19), and 85% of cT4 patients (*n* = 13) tumors at baseline. For the patients who experienced pathologic downstaging, the median tumor size was 9.3 cm as assessed on imaging at baseline, 6.9 cm as assessed on imaging preoperatively, and 6.0 cm as assessed by pathology at resection.

**Table 4.** Characteristics of patients who experienced pathologic complete response.

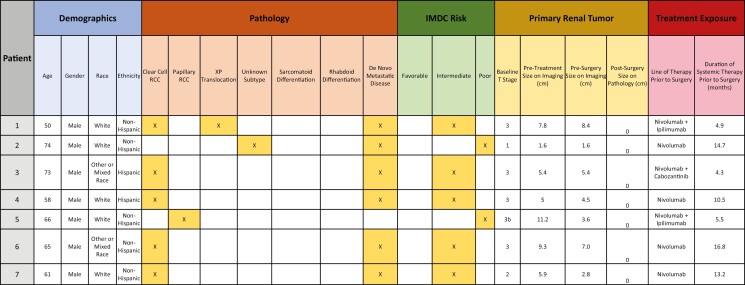

It was also notable that seven (13%) patients had no residual disease (ypT0) in their renal primary at CN. The heterogeneous characteristics of these extreme responders are illustrated in Table 4. Among patient achieving a complete response in the primary tumor, baseline cT stages (maximal diameter) were 14% cT1 (*n* = 1) (1.6 cm), 14% cT2 (*n* = 1) (10.1 cm), and 71% cT3 (*n* = 5) (7.8, 5.4, 5.0, 11.2, 9.3 cm). Pathologic subtypes included clear cell in 5 patients, papillary RCC in one, and XP translocation RCC in one. None of these tumors demonstrated sarcomatoid or rhabdoid differentiation. Six patients had distant metastases at diagnosis, and one had locally advanced disease at baseline.

In addition, 8 (15%) patients had ypT1 disease at CN, of whom 4 patients had T stage downstaging from their cT stage measured at baseline. Of the 4 patients who experienced downstaging to ypT1, 75% (*n* = 3) had cT2 disease at baseline and 25% (*n* = 1) had cT4 disease at baseline. All patients with ypT1 at CN had residual tumors at were at least 3 cm in length based on surgical pathology.

Pathologic outcomes at the time of CN demonstrated median tumor size 6.5 cm with 85% (*n* = 42) of patients having negative margins and 75% (*n* = 39) with necrosis.

### Imaging Response

The overall ORR (including renal and extra-renal metastatic lesions) immediately prior to nephrectomy was 53.8% (*n* = 28): 63% partial response (*n* = 33), 29% stable disease (*n* = 15), 4% progressive disease (*n* = 2), and 4% unknown (*n* = 2). The RECIST response rates for the renal primary tumor immediately prior to nephrectomy were 42% partial response (*n* = 22), 40% stable disease (*n* = 21), 10% progressive disease (*n* = 5), and 8% unknown (*n* = 4). This is tabulated in Table 2.

### Survival

**Figure 2.**
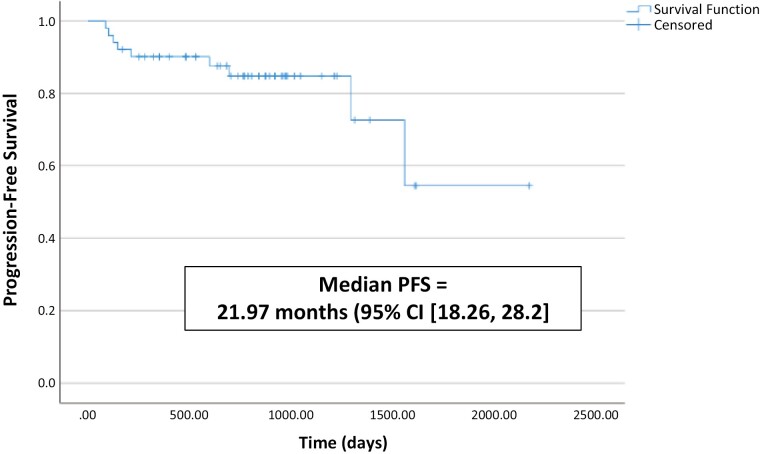
Progression-free survival for the entire cohort.

Median follow-up for the entire cohort was 25.3 months. The median PFS for the entire cohort was 3.5 years (95% CI, 2.1-4.87, Fig. 2). The 2-year PFS was 76.5% and 3-year PFS was 63.7%. The study population was divided into 2 groups: those with (*n* = 23) and without (*n* = 29) ypT downstaging at time of CN. The 3-year PFS for patients with versus without ypT downstaging at time of CN was 86.1% versus 84.2%, respectively (*P* = .749). There were 3 deaths reported during follow-up, including one in a patient who had experienced downstaging. All 3 deaths occurred in pT3a patients and occurred due to disease progression of RCC. Given limited number of deaths, overall survival was not reported.

## Discussion

In the modern immunotherapy era, the data supporting the role of CN for patients with metastatic RCC is limited. In this work, we examined the pathologic outcomes of patients with advanced or metastatic RCC at CN following IO therapy. We demonstrated that 44% of patients experienced pathologic T downstaging with a median 17.8% reduction in renal tumor size by pathology assessment compared to baseline imaging. Furthermore, 13% (*n* = 7/52) of patients had no residual tumor at CN.

To date, in the trials establishing the superior efficacy of IO combinations over VEGF monotherapy in metastatic RCC, the majority of the enrolled patients underwent nephrectomy prior to systemic therapy initiation. In the landmark frontline studies of IO combination therapies in RCC, there has been a decline in CN utilization, reflective of changes in practice patterns in the post-CARMENA era: rates of baseline CN were 81% for CheckMate 214, 83% for KEYNOTE 426, 80% for Javelin Renal 101, 69% for CheckMate 9ER, 73% for the CLEAR study and in the COSMIC-313 study of triplet therapy with nivolumab, ipilimumab, and cabozantinib the CN rate was 65%.^,14-18^ A subgroup analysis of 108 patients who did not undergo CN from the CheckMate 214 trial demonstrated an ORR of 35% with nivolumab plus ipilimumab; however, no complete responses were observed in this population.

Studies that have analyzed and supported the combination of IO with surgical resection of the primary tumor have been conducted in other solid tumor types, including lung and melanoma, but these data are lacking in RCC.^20-22^ Thus far, current data have been mostly derived from case reports and retrospective studies, which demonstrate enhanced efficacy of IO prior to CN in the metastatic disease.^23-26^ A retrospective analysis of 391 patients with advanced RCC in the National Cancer Database demonstrated superior OS in patients that received both IO and CN compared to IO alone. Within this study, 10% (*n* = 2/20) patients who received IO prior to their CN had a complete pathologic response.^27^ Another retrospective analysis from IMDC Consortium evaluated 43 patients who received IO followed by CN (*n* = 142) and demonstrated improved OS associated with CN compared to patient who received IO and did not undergo CN (*n* = 55) (hazard ration (HR) = 0.39 [0.19-0.83]).^28^ An institutional case series of 10 patients with advanced RCC who received nivolumab and ipilimumab prior to CN demonstrated that CN provided favorable pathologic outcomes: one individual experiencing a complete pathologic response and 3 patients rendered to have a completed response post metastatectomy.^29^

A national retrospective study performed in France assessed disease-free survival (DFS), PFS, and OS in 30 patients with metastatic RCC who received IO with either PR or CR prior to nephrectomy. The median duration of IO treatment prior to surgery was 10 months and this led to a PFS of 96.7% and OS of 100% at 1 year, and 78.3% and 86.1% at 2 year, respectively.^30^ Additionally, there have several prospective trials investigating nephrectomy following IO in the non-metastatic setting. A single center study analyzed feasibility and safety of 18 locally patients with advanced RCC who received nephrectomy after 4, 2-week cycles of nivolumab. Results showed that 16/18 patients received all doses of nivolumab prior to nephrectomy. For all patients, the best imaging response in the primary tumor to IO was stable disease. When comparing the pre-IO and post-IO biopsy specimens, there was at least a 5% tumor regression in 10/14 evaluable cases.^31^ Another small prospective study evaluated the safety and tolerability of 17 non-metastatic patients who received nephrectomy following 3, 2-week cycles of nivolumab. Of these patients, all had stable disease except for one patient who showed features of an immune-related pathologic response.^32^

The timing and candidacy for patients who receive delayed nephrectomy is still under investigation in the modern era. Due to the retrospective and multi-institutional nature of our study, there was variability in the selection criteria for individuals who were candidates for CN, as this was clinician dependent, and timing of CN relative to receipt of anti-cancer therapy. A retrospective study utilizing the IMDC database of patients with de novo metastatic RCC who received upfront CN demonstrated significantly higher OS in patients having received CN compared to those receiving systemic therapy alone (hazard ratio [HR]: 0.61; 95% CI [CI], 0.41-0.90, *P* = .013). The median duration of time between CN and immunotherapy initiation was 2.5 months. From the multi-variable analysis (MVA) performed in this study, older patients (>65 years), low performance status, and presence of certain metastatic sites (bone, brain, liver) were less likely to be selected for CN.^33^ Another study sought to determine modifiable IMDC risk factors and outcomes present in 245 metastatic RCC patients referred for CN. Patients receiving CN had fewer IMDC risk factors (*P* = .003), fewer metastases (*P* = .011), and higher rates of clear cell histology (*P* = .012). These data highlight that CN is still a viable treatment options in select patients with advanced RCC. While selection criteria for upfront or delayed CN are lacking, typically patients with less disease burden and comorbidities seems to derive the greatest benefit with this approach.

In terms of limitations for our study, it is important to note the potential for selection bias present in our analysis given the retrospective nature of this work. The cohort is skewed toward patients deemed healthy enough to be candidates for CN by clinicians at high-volume academic centers, which may have implications for generalizability of the results. Additionally, overall survival was not reported due to short follow-up time and limited number of deaths in the cohort. The sample size of patients was small, and this was a single arm study with no comparator. The small sample size made it difficult to perform an MVA. Subgroup analyses and conclusions are limited by the heterogeneity in systemic therapy combinations used including regimens and doses/duration of IO received. Given retrospective nature of the analysis, we were not able to assess intent (for cytoreduction or palliation) and difficulty of the operative procedure. Our dataset did not include patients who received IO therapy and experienced a response, either CR or PR, and did not undergo surgical resection. Lastly, repeat pathologic assessment was performed only on patients who experienced a complete pathologic response. Despite these limitations, our analysis does provide preliminary data for larger prospective trials that are investigating the impact of IO prior to nephrectomy.

Currently, 2 prospective clinical trials (NORDIC-SUN [NCT03977571] and Southwestern Oncology Group (SWOG) 1931/PROBE [NCT04510597]) are evaluating the role of CN in the modern era with upfront IO-based treatment.^34^ These studies will be important in helping define the role of CN in the modern immunotherapy area.

As the field moves forward in optimizing therapy selection for patient with advanced RCC, it will be critically important to understand molecular predictors of favorable response to IO-based treatment. Candidate biomarkers have been plentiful including PD-1/PD-L1 tumor status, *PBRM1* mutation status, T-cell infiltration, and ribonucleic acid (RNA) gene signatures; however, currently no biomarker is currently used to aid in therapy selection for patients.^35-38^ Future genomics studies evaluating molecular predictors of response and the tumor microenvironment could further illuminate predictive biomarkers to inform IO therapy selection for patients with advanced or metastatic RCC.^36^ The utilization of genomics for clinical decision-making is being studied in the OPTIC trial (NCT05361720), which is currently analyzing the effects of front-line systemic therapy assignment (either nivolumab/cabozantinib or ipilimumab/nivolumab), based on RNA sequence defined biologic cluster, on patient ORR.

## Conclusion

Our study demonstrates that CN following IO is feasible. After receipt of IO, pathological downstaging occurred in the renal primary tumor in a subset of patients in our cohort. Future analyses to elucidate molecular predictors of response and resistance to IO therapy from paired tissue samples derived from baseline biopsy and nephrectomy are underway.

## Data Availability

The data underlying this article will be shared on reasonable request to the corresponding author.
